# A Genome-Wide Association Study Exploring the Genetic Architecture of Eggshell Quality in Chinese Wenshang Barred Chickens

**DOI:** 10.3390/ani16142157

**Published:** 2026-07-11

**Authors:** Jie Wang, Haixia Han, Jie Liu, Qiuxia Lei, Wei Liu, Yan Sun, Dan Hao, Fuwei Li, Xinxia Wang, Dingguo Cao, Xinhua Zhao, Yan Zhou

**Affiliations:** 1 Poultry Institute, Shandong Academy of Agricultural Sciences, Jinan 250100, China; wj_saas@163.com (J.W.); hanhaixia@163.com (H.H.); jqsyzslj@163.com (J.L.); lei_qiuxia@163.com (Q.L.); lwteam@126.com (W.L.); sunyan881102@163.com (Y.S.); haodan111121@163.com (D.H.); lifuwei1224@163.com (F.L.); cdgjqs@163.com (D.C.); jqskyb@163.com (X.Z.); 2 Jinan Key Laboratory of Poultry Germplasm Resources Innovation and Healthy Breeding, Jinan 250100, China; 3 Shandong Provincial Key Laboratory of Livestock and Poultry Breeding, Jinan 250100, China; 4 Lijin Center for Animal Disease and Control, Dongying 257091, China; wxx13864729676@163.com

**Keywords:** laying hens, eggshell quality, GWAS, SNP, Wenshang Barred chicken

## Abstract

Eggshell quality is a critical trait in poultry production, as it directly influences economic outcomes. The genetic architecture underlying this trait remains poorly characterized. In this study, we conducted a genome-wide association study (GWAS) on Chinese Wenshang Barred chickens at 43 weeks of age to identify genomic loci and candidate genes associated with eggshell quality. This analysis revealed 87 significant SNPs and 14 candidate genes. Genes including *MC2R*, *CDC73*, *LYSMD2*, *TNFAIP8L3*, and *CYP19A1* were associated with eggshell weight; *PHEX*, *ATM*, *STXBP6*, *SLIT3*, and *RALY* with eggshell thickness; and *SOCS5* and *ATG4C* with eggshell strength. Notably, three SNPs emerged as the most promising markers for eggshell quality, though their functional roles in laying hens require further investigation. This study advances our understanding of the genetic basis of eggshell quality and holds significant practical implications for improving this trait through targeted breeding strategies.

## Simple Summary

Eggshell quality is one of the most economically important traits in poultry production, as it profoundly affects the production efficiency and economic returns of the laying industry. Previous studies have predominantly focused on commercial chicken lines, whereas the molecular genetic regulatory mechanisms governing eggshell quality remain poorly characterized in Chinese indigenous chickens. To address this research gap, our study set out to perform a genome-wide association study (GWAS) on 43-week-old Wenshang Barred chickens to identify pivotal genetic variations and functional genes associated with eggshell quality traits. Overall, 87 molecular markers and 14 functional candidate genes associated with eggshell weight, eggshell thickness, and eggshell strength were identified in this population. It is worth noting that three outstanding genetic markers emerged as the most promising markers for eggshell quality for future breeding use, while their detailed functional mechanisms need further experimental validation. These findings enrich the genetic theoretical basis of eggshell trait formation in native chickens and provide valuable molecular markers for the targeted breeding of superior eggshell quality. In practical production, the identified markers can be utilized for MAS targeting eggshell quality in indigenous chickens to reduce egg breakage losses and improve the production value of indigenous chicken breeds.

## 1. Introduction

Eggshell quality constitutes a critical economic trait in poultry production, directly impacting the profitability of egg and hatchery industries. Substandard shell quality increases susceptibility to breakage during handling, transportation, and storage, resulting in significant economic losses and diminished marketability [1,2]. The structural integrity of the eggshell is determined by its ultrastructural organization, which comprises three distinct layers: the mammillary layer originating from nucleation sites, the palisade layer consisting of columnar crystals, and the vertical crystal layer representing the terminal stage of calcification [3,4]. Eggshell formation occurs primarily in the hen’s uterus (eggshell gland) over approximately 10 to 22 h, representing the longest stage of the egg-forming process [5]. Research has demonstrated that the density, size, and thickness of mammillary knobs, along with the palisade layer’s thickness, directly influence mechanical properties such as strength, thickness, and overall shell quality [6]. Disruptions in this meticulously coordinated mineralization process can lead to structural defects, manifesting as cracks, translucency, or surface irregularities.

The factors contributing to eggshell quality variation are multifaceted, encompassing nutritional, environmental, health-related, and genetic determinants [7]. Given the complex etiology of eggshell quality variation, genetic improvement through selective breeding represents a sustainable approach to enhancing this economically important trait. The heritability of eggshell quality traits has been estimated in various populations, with values ranging from moderate to high (reported h^2^ values of approximately 0.20–0.60 for eggshell weight, thickness, and strength), indicating that genetic selection could be effective [8,9,10]. Genome-wide association studies (GWASs) have emerged as powerful tools for dissecting the genetic basis of complex traits in livestock species [11,12]. In chickens, GWAS approaches have been successfully employed to identify genomic regions and candidate genes associated with various egg quality traits [8]. For instance, high-density SNP arrays have revealed significant associations on chicken chromosome 13 for albumen height and Haugh units, with the *MSX2* gene and *DRD1* gene proposed as candidate genes [13]. Similarly, genomic regions on chromosome 1 have been implicated in eggshell strength, with the *FRY* gene and *PCNX2* gene identified as potential candidates in Rhode Island Red chickens [8]. Studies on eggshell speckles have further uncovered 39 candidate genes involved in calcium transport, phospholipid metabolism, and immunoregulation, including *SPTBN5*, *EHD4*, and members of the *PLA2G4* family [14]. More recently, a CNV-based GWAS in Wenshui green shell-laying chickens highlighted genes such as *FAM184B*, *MED28*, and *LAP3* as potential contributors to egg quality traits [15]. While these studies have significantly advanced the understanding of the genetic determinants of eggshell quality, most research has focused on commercial lines or specific breeds, leaving the genetic basis of this trait in indigenous Chinese poultry populations largely understudied.

This study aimed to identify genomic loci and candidate genes associated with eggshell quality traits in Wenshang Barred chickens at 43 weeks of age through a genome-wide association study (GWAS). By characterizing single-nucleotide polymorphisms (SNPs) and their contributions to phenotypic variation in eggshell weight, thickness, and strength, we sought to uncover the genetic basis of eggshell quality in this indigenous chicken breed. The findings will not only advance the understanding of the molecular mechanisms underlying eggshell formation but also provide potential molecular markers for marker-assisted selection to enhance eggshell quality. These results contribute to the broader knowledge of eggshell genetics and support the sustainable utilization of indigenous chicken genetic resources.

## 2. Materials and Methods

### 2.1. Resource Population and Phenotype Collection

A total of 449 Chinese Wenshang Barred (WB) chickens were sourced from Jinqiu Agriculture and Animal Husbandry Co., Ltd. (Jining Shandong, China). Phenotypic measurements were performed at 43 weeks of age, corresponding to the peak laying period. For each hen, three consecutive eggs were collected within a 3-day window, and the mean value was used as the individual phenotypic record. All eggs were measured within 24 h of collection to ensure the consistency of storage conditions. Eggshell weight (ESW) was measured using a digital electronic balance (Ohaus Corporation, Parsippany, USA) after the egg contents were removed, the shells were dried and cleaned, and the inner membrane was carefully excised. Eggshell fragments were then sequentially separated into three anatomical regions: the blunt end, middle section, and sharp end. Eggshell thickness (EST) was quantified using a digital thickness gauge (ORKA Food Technology Ltd., Ramat HaSharon, Israel), while eggshell strength (ESS) was assessed with a mechanical strength tester (ORKA Food Technology Ltd., Ramat HaSharon, Israel). Phenotypic data for all traits were found to follow a normal distribution within the range of the mean ± 3 standard deviations and met quality control criteria for downstream statistical analysis.

### 2.2. Genotyping and Quality Control

Blood samples were collected from the wing vein (Supplementary Table S1), and genomic DNA was extracted using phenol–chloroform extraction. DNA quality was evaluated via agarose gel electrophoresis prior to library preparation, followed by the construction of 2 × 150 bp paired-end sequencing libraries. Sequencing was conducted on the DNBSEQ platform (BGI Genomics, Shenzhen, China). Raw sequence data were processed using SOAPnuke (v1.5.6) to remove adapter sequences and low-quality reads. Clean reads were aligned to the chicken reference genome (GRCg6a, Ensembl release 106; URL: https://ftp.ensembl.org/pub/release-106/fasta/gallus_gallus/dna/, accessed on 10 may 2025) using Burrows–Wheeler Aligner (BWA) algorithm and sorted with Samtools v1.2. Read mapping quality was assessed with Qualimap 2, and reads with a quality score < 30 were excluded. Single-nucleotide polymorphisms (SNPs) were identified using GATK HaplotypeCaller v3.3 under stringent filtering parameters: QD < 2.0, FS > 60.0, RMSMapQ < 40.0, MQRankSum < –12.5, HaplotypeScore > 13.0, and ReadPosRankSum < –8.0. After filtering for MAF < 0.01 and SNP call rate < 0.05, 9,427,781 SNPs across 28 autosomes were retained for analysis.

### 2.3. Genome-Wide Association Study for Eggshell Quality in Wenshang Barred Chicken

To elucidate the genetic architecture of eggshell quality traits (eggshell weight, ESW; shell thickness, EST; and shell strength, ESS), a genome-wide association study (GWAS) was conducted using a linear mixed model implemented in GEMMA (v0.98.4) on whole-genome sequencing data from chickens. The statistical model was formulated as follows:

(1) y=Wα+Xβ+u+e

where **y** represents the vector of phenotypic observations, **W** denotes the covariate matrix (including a column of 1 for the intercept), **α** is the vector of coefficients for fixed effects, X is the genotype matrix for single-nucleotide polymorphisms (SNPs), **β** represents the effect size of each SNP, **u** accounts for random polygenic effects, and **e** is the residual error term. The fixed effects included in the covariate matrix **W** consisted of the first five principal components (PCs) to correct for population stratification, along with the intercept. No additional batch, cage, or family effects were explicitly incorporated, as the study population was maintained under uniform housing and management conditions within a single flock. Linkage disequilibrium (**LD**) pruning was applied using a 25 SNP sliding window with a 5 SNP step size and an r^2^ threshold of 0.2, resulting in 847,530 independent SNPs. Genome-wide and suggestive significance thresholds were established via Bonferroni correction (0.05/847,530 and 1/847,530, respectively). Manhattan and quantile–quantile (Q-Q) plots were generated using the CMplot package in R v4.5.2 to visualize associations and assess statistical validity.

Using the chicken GRCg6a genome, the positions and information of SNPs in the GWAS results were retrieved. SnpEff software (v5.3a) [16] was then used to perform gene annotation for the significantly associated SNPs, with an annotation window of 5000 bp upstream and downstream. The identified genes were considered candidate genes.

### 2.4. Phenotypic Variation Explained

The phenotypic variation explained (PVE) was calculated using the following formula:

(2) $$PVE=\frac{2\beta^{2}MAF\left(1-MAF\right)}{2\beta^{2}MAF\left(1-MAF\right)+(se\left(\beta \right){)}^{2}2NMAF\left(1-MAF\right)}$$

where β represents the effect value from the GWAS results, MAF denotes the minimum allele frequency of the SNP, and N represents the number of individuals involved in the GWAS analysis.

### 2.5. Further Analysis of QTL Regions

Region-based association analysis represents an efficient method for pinpointing causal single-nucleotide polymorphisms (SNPs). To further explore significant and physically adjacent SNPs linked to traits of interest, regional association analyses were conducted using the IntAssoPlot package in R v4.5.2.

### 2.6. Statistical Analysis

Statistical analysis was performed using IBM SPSS Statistics for Windows, Version 25.0 (IBM Corporation, Armonk, NY, USA), and GraphPad Prism 9.

## 3. Results

### 3.1. Data Characteristics

**Figure 1:**
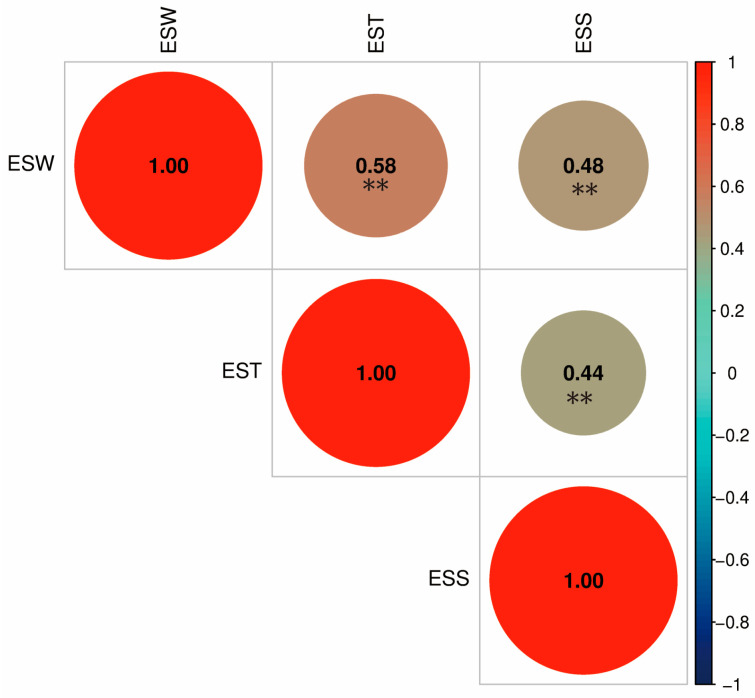
Correlation analysis of ESW, EST and ESS traits. * *p* < 0.05; ** *p* < 0.01.

The eggshell quality traits of WB hens at 43 weeks of age are summarized in Table 1 (*n* = 449). The mean ESW was 5.01 g (SD = 0.46 g), mean EST was 0.41 mm (SD = 0.05 mm), and mean ESS was 39.79 N/cm^2^ (SD = 7.10 N/cm^2^). The lowest variability (smallest CV) was observed for eggshell weight (9.20%) and eggshell thickness (11.44%), indicating relatively uniform trait expression. The highest variability (largest CV) was recorded for eggshell strength (17.84%), reflecting greater phenotypic diversity in this trait. Eggshell strength showed the greatest absolute range of variation (15.91–55.89 N/cm^2^). The phenotypic traits exhibit adequate phenotypic variation to support subsequent GWAS analysis. Additionally, significant moderate positive correlations were observed among eggshell weight (ESW), eggshell thickness (EST), and eggshell strength (ESS) (r = 0.44–0.58, *p* < 0.01). (Figure 1).

### 3.2. Principal Component Analysis (PCA)

**Figure 2:**
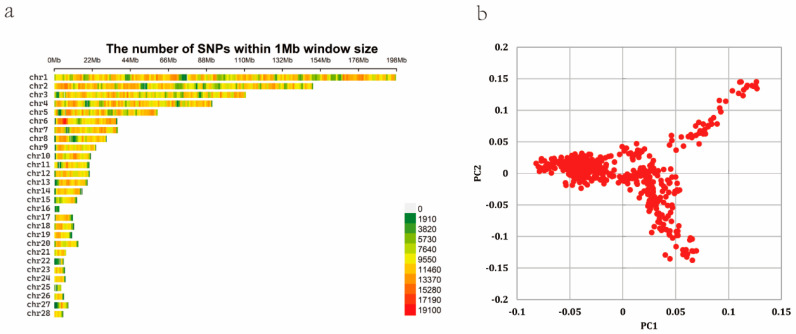
Population genomic features. (**a**) Distribution of SNP density across 28 chromosomes. (**b**) PCA plot of WB chicken breed.

The distribution of SNP density across the 28 chromosomes of the WB chicken population, along with genetic stratification inferred from PCA, is presented in Figure 2. The population formed three distinct clusters, indicating significant genetic structure within the reference group. Most individuals clustered in the central region, with a minority dispersed across other regions. To mitigate the effects of population stratification on association analyses, the first five principal components were incorporated as covariates (selected based on scree plot analysis showing a clear inflection point after PC5, together with their eigenvalue contributions, as shown in Figure 2b).

### 3.3. GWAS Analysis and Gene Function Annotation of ESW Trait

**Figure 3:**
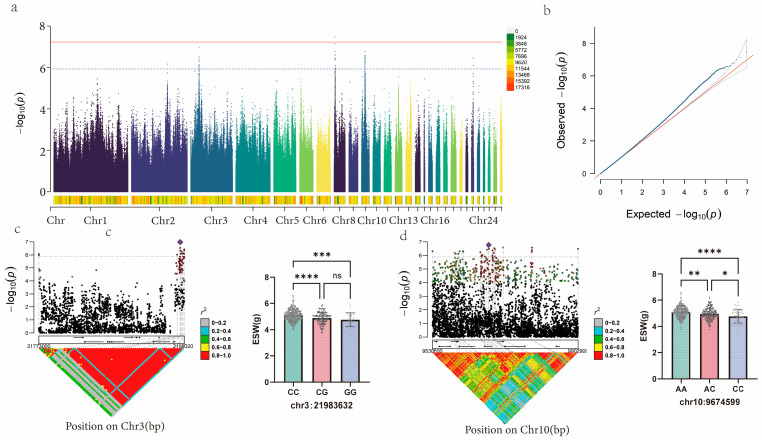
The GWAS results of ESW. (**a**) Manhattan plot; (**b**) QQ plots; (**c**,**d**) regional association plot for significant SNPs. In the Manhattan plot, the X-axis represents the chromosomal position corresponding to each SNP. The Y-axis represents -log10 (*p* values). The red and blue lines represent the genome-wide significant threshold (5.90 × 10^−8^) and suggestive significant threshold (1.18 × 10^−6^), respectively. The QQ plot shows the expected -log10-transformed *p* value against the observed -log10-transformed *p* value. * *p* < 0.05; ** *p* < 0.01; *** *p* < 0.001; **** *p* < 0.0001.

The GWAS Manhattan and Q-Q plots for eggshell weight (ESW) are presented in Figure 3a,b. The Q-Q plots show most data points aligning closely with the expected distribution, indicating that population structure was effectively accounted for in the analysis, and thus, no inflation or systematic bias was detected. The Manhattan plots identified 57 significant SNPs associated with ESW on chromosomes (GGA) 3, 8, and 10. These SNPs were located near candidate genes potentially involved in ESW, including *MC2R*, *CFH*, *CDC73*, *LYSMD2*, *TNFAIP8L3*, and *CYP19A1*. Detailed SNP information is summarized in Table 2, while supplementary data are provided in Supplementary Tables S2 and S3. Region-based association plots for GGA3 and GGA10, which encompass multiple SNPs within linkage disequilibrium (LD) blocks, are displayed in Figure 3c,d. On GGA3 (21.77-21.99 Mb; 13 SNPs), the most significantly associated SNP, located at position 3:21983632, showed a strong link to ESW. LD analysis revealed moderate LD levels across several haplotype blocks (Figure 3c). Notably, individuals with the CC genotype exhibited significantly higher ESW compared to those with the GG genotype (Figure 3c and Table 3). On GGA10 (9.53-9.90 Mb; 26 SNPs), the leading SNP, positioned at 10:9674599 near the *CYP19A1* gene, was strongly associated with ESW. Individuals with the AA genotype demonstrated significantly greater ESW than those with the CC genotype (Figure 3d and Table 3).

### 3.4. GWAS Analysis of EST Trait

**Figure 4:**
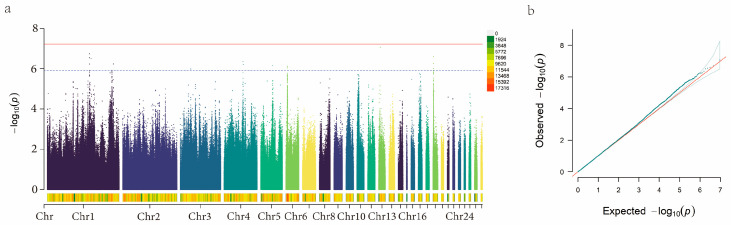
The GWAS results of EST. (**a**) Manhattan plot; (**b**) QQ plots. In the Manhattan plot, the X-axis represents the chromosomal position corresponding to each SNP. The Y-axis represents −log10 (*p* values). The red and blue lines represent the genome-wide significant threshold (5.9 × 10^−8^) and suggestive significant threshold (1.18 × 10^−6^), respectively. The QQ plot shows the expected −log10-transformed *p* value against the observed −log10-transformed *p* value.

The GWAS Manhattan and Q-Q plots for eggshell thickness (EST) are presented in Figure 4a,b. The Manhattan plots identified 18 significant SNPs associated with EST on GGA1, GGA3, GGA4, GGA5, GGA6, GGA13, and GGA20. Candidate genes potentially involved in EST regulation include *DMD*, *PHEX*, *ATM*, *STXBP6*, *SLIT3*, and *RALY* (Table 2).

### 3.5. GWAS Analysis and Gene Function Annotation of ESS Trait

**Figure 5:**
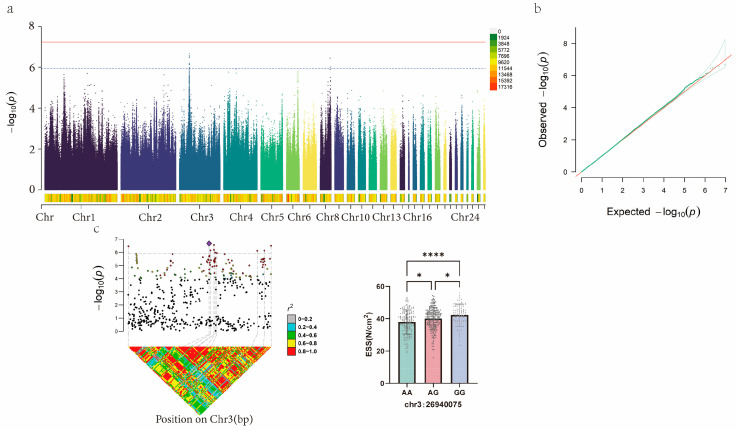
The GWAS results of ESS. (**a**) Manhattan plot; (**b**) QQ plots; (**c**) regional association plot for significant SNPs. In the Manhattan plot, the X-axis represents the chromosomal position corresponding to each SNP. The Y-axis represents −log10 (*p* values). The red and blue lines represent the genome-wide significant threshold (5.90 × 10^−8^) and suggestive significant threshold (1.18 × 10^−6^), respectively. The QQ plot shows the expected −log10-transformed *p* value against the observed −log10-transformed *p* value. * *p* < 0.05; ** *p* < 0.01; *** *p* < 0.001; **** *p* < 0.0001.

The GWAS Manhattan and Q-Q plots for eggshell strength (ESS) are presented in Figure 5a,b. Most points clustered near the diagonal line in the Q-Q plots, indicating the absence of genomic inflation or systematic bias due to the incorporation of population structure into the GWAS analysis. The Manhattan plots identified 12 significant SNPs associated with ESS on GGA3, GGA8, and GGA20. Genes implicated in eggshell strength include *SOCS5* and *ATG4C* (Table 2, Supplementary Tables S2 and S3). A region-based association plot for GGA3, which includes SNPs in an LD-associated region, is shown in Figure 5c. The strongest associated SNP in this region (26.91–26.96 Mb, 10 SNPs) is located at position 3:26940075 within the *SOCS5* gene. LD analysis revealed moderate linkage disequilibrium levels across several haplotype blocks (Figure 5c). We observed that individuals with the GG genotype exhibited greater eggshell strength compared to those with the AA genotype (Figure 5c and Table 3).

## 4. Discussion

In this study, we conducted a GWAS on Chinese Wenshang Barred chickens at 43 weeks of age to elucidate the genetic architecture of eggshell quality traits, including eggshell weight (ESW), eggshell thickness (EST), and eggshell strength (ESS). We identified 87 significant SNPs distributed across multiple chromosomes and annotated 14 candidate genes potentially involved in regulating these traits. Our findings provide novel insights into the molecular mechanisms underpinning eggshell quality variation in this indigenous Chinese breed.

The Q-Q plots for all three traits showed minimal inflation, with most observed *p* values closely following the expected distribution, indicating that the inclusion of the first five principal components as covariates effectively controlled for population stratification. This is consistent with the complex genetic structure detected by PCA, which revealed three distinct clusters within the WB population, reflecting its diverse genetic background. Previous GWASs on eggshell quality have similarly reported that accounting for population stratification is critical to reduce false-positive associations [8,9]. The moderate-to-high coefficient of variation observed for ESS (17.84%) compared to ESW (9.20%) and EST (11.44%) suggests that eggshell strength is subject to greater environmental and genetic influence, consistent with prior reports [8].

In this study, we identified that genes such as *MC2R*, *CDC73*, *CYP19A1*, *TNFAIP8L3*, *DMD*, *PHEX*, *ATM*, *SLIT3*, *RALY*, *SOCS5*, and *ATG4C* were associated with eggshell quality. Our findings partially overlap with and extend previous GWASs on eggshell quality in chickens. Chen et al. (2024) identified *FRY* and *PCNX2* as candidate genes for eggshell strength in Rhode Island Red chickens on GGA1, while our study identified *SOCS5* and *ATG4C* on GGA3 and GGA8, respectively, highlighting the genetic heterogeneity of eggshell strength between breeds [8]. Sun et al. (2015) reported promising QTL regions on GGA4 and GGA13 for eggshell quality in an F2 resource population [9], and we similarly detected signals on GGA13 (*SLIT3*) for EST. Liu et al. (2018) identified *MSX2* and *DRD1* as candidate genes on GGA13 for Haugh units in a late-laying population [13].

In this study, *MC2R*, *CDC73*, *CYP19A1*, *LYSMD2* and *TNFAIP8L3* were identified as candidate genes related to eggshell weight. MC2R (Melanocortin 2 Receptor) encodes the adrenocorticotropic hormone (ACTH) receptor and plays a central role in the hypothalamic–pituitary–adrenal (HPA) axis by mediating ACTH-stimulated glucocorticoid biosynthesis [17,18]. The dietary supplementation of corticosterone has been shown to significantly affect eggshell thickness and reduce production performance in laying hens [19]. *MC2R*’s role in regulating glucocorticoid secretion and the downstream effects on calcium absorption and transport suggest a plausible biological pathway connecting *MC2R* activity to eggshell weight. CDC73 (Cell Division Cycle 73) is associated with parathyroid tumors and hyperparathyroidism–jaw tumor (HPT-JT) syndrome, characterized by severe hypercalcemia due to dysregulated parathyroid hormone (PTH) secretion [20]. Given that PTH is a master regulator of calcium homeostasis and directly influences bone calcium mobilization and renal calcium reabsorption—processes critical for eggshell calcification—*CDC73* may indirectly modulate calcium availability for eggshell formation. CYP19A1 (Cytochrome P450 Family 19 Subfamily A Member 1) is responsible for the conversion of androgens to estrogens. *CYP19A1* plays a critical role in female reproductive physiology and gonadal differentiation in chickens [21]. Estrogens are essential for the formation of medullary bone, the primary calcium reservoir mobilized during eggshell calcification in laying hens [22]. Furthermore, *CYP19A1* polymorphisms have been significantly associated with bone mineral density in postmenopausal women [23,24], reinforcing the role of aromatase-derived estrogen in calcium metabolism. In the context of laying hens, adequate estrogen signaling is essential to maintain the medullary bone calcium pool that supports eggshell formation. Variation in *CYP19A1* expression or activity could therefore affect estrogen availability, medullary bone dynamics, and ultimately eggshell weight. TNFAIP8L3 belongs to the TIPE (tumor necrosis factor-α-induced protein 8-like) family, which regulates inflammation and tumorigenesis [25]. Inflammatory signaling has been implicated in the modulation of calcium transport and eggshell quality in the uterus [26], suggesting that the *TNFAIP8L3*-mediated regulation of inflammatory pathways may influence eggshell calcification.

*DMD*, *PHEX*, *ATM*, *STXBP6*, *SLIT3* and *RALY* were identified as candidate genes related to eggshell thickness. DMD (Dystrophin) is a structural cytoskeletal protein critical for maintaining the integrity of muscle cell membranes, and *DMD* mutations have been associated with reduced bone mineral density in affected patients [27]. PHEX (Phosphate-Regulating Endopeptidase) encodes a zinc metallo endopeptidase that regulates phosphate metabolism and bone mineralization by cleaving the ASARM peptide derived from MEPE [28]. The PHEX-FGF23-MEPE-ASARM axis is critical for the coordination of bone mineralization and phosphate homeostasis [28,29]. Given that eggshell calcification depends on both calcium and phosphate ion availability and the activity of matrix proteins, *PHEX* may regulate the phosphate environment within the shell gland, thereby influencing eggshell thickness. *ATM* (Ataxia Telangiectasia Mutated) has been recognized as a metabolic regulator involved in oxidative stress response and cellular energy homeostasis [30]. Increased oxidative stress has been associated with impaired eggshell quality in laying hens [31], and the *ATM*-mediated regulation of the cellular redox state may influence the oxidative microenvironment of the shell gland during eggshell calcification. *STXBP6 *(Syntaxin-Binding Protein 6) encodes a negative regulator of vesicle fusion through interaction with the SNARE complex. Vesicular transport and exocytosis are fundamental mechanisms by which calcium and calcium-binding proteins are secreted into the eggshell calcification milieu in the uterus [32,33]. SLIT3 was identified as a candidate gene for EST on GGA13. *SLIT3* encodes a secreted protein of the Slit family of axon guidance molecules. Importantly, osteoclast-secreted *SLIT3* has been shown to act as a key coupling factor that simultaneously promotes osteoblast migration and proliferation (enhancing bone formation) while suppressing osteoclast differentiation (reducing bone resorption) through ROBO1/ROBO2-mediated β-catenin signaling [34]. The loss of *SLIT3* in mice resulted in osteoporosis due to uncoupled bone remodeling, and higher circulating *SLIT3* levels were correlated with greater bone mineral density in postmenopausal women [34]. *SLIT3* may play a similar coupling role in the shell gland, coordinating matrix degradation and calcium deposition during eggshell formation. RALY functions as an RNA-binding protein involved in transcriptional regulation, particularly of cholesterol biosynthetic genes [35].

*SOCS5* and *ATG4C* were identified as candidate genes related to eggshell strength. The most significant finding for ESS was on GGA3, with the lead SNP located within the *SOCS5* gene. SOCS5 (suppressor of cytokine signaling 5) encodes a member of the suppressor of cytokine signaling (SOCS) protein family, which acts as a classical negative feedback regulator of cytokine signal transduction through the JAK/STAT pathway and epidermal growth factor (EGF) signaling [36]. Individuals carrying the AA genotype at the lead SNP (3:26940075) showed significantly higher eggshell strength than those with the GG genotype. JAK/STAT cytokine signaling pathways are known modulators of osteoblast and osteoclast activity during bone remodeling [37]. Moreover, EGF signaling regulates the proliferation and secretory activity of uterine epithelial cells in the shell gland [38]. The dysregulation of EGF signaling through altered SOCS5 activity may therefore affect the deposition of calcite crystals and the mechanical properties of the eggshell. ATG4C (Autophagy-Related 4C Cysteine Peptidase) encodes a cysteine protease involved in the processing and lipidation of ATG8 family proteins required for autophagosome biogenesis [39]. In the context of eggshell formation, autophagy in shell gland cells may regulate the turnover of calcium transport machinery and matrix proteins, ultimately affecting mechanical strength.

The phenotypic variation explained (PVE) by the three lead SNPs ranged from 5.83% to 6.12%, indicating that these loci each exert a modest but biologically meaningful effect on the respective traits. Such effect sizes are typical for quantitative traits in livestock GWASs [8,9], supporting a polygenic architecture for eggshell quality in WB chickens. Three SNPs were identified as the most promising markers associated with eggshell quality: SNP at 3:21983632 (ESW, CC > GG), rs80641237 at 10:9674599 (ESW, AA > CC), and rs731402681 at 3:26940075 (ESS, AA > GG). Each SNP explained approximately 5.83% to 6.12% of phenotypic variance. The LD analysis confirmed that these SNPs are located within distinct haplotype blocks associated with their respective traits, and no significant interaction effect was detected between the two ESW lead SNPs on GGA3 and GGA10. These SNPs may serve as useful molecular markers for marker-assisted selection (MAS) aimed at improving eggshell quality in WB chickens, and the integration of these SNPs into genomic prediction models could also improve the accuracy of breeding value estimation for eggshell quality in Chinese indigenous breeds. However, the validation of these markers in independent populations and functional characterization of the causal variants are warranted before their implementation in breeding programs.

Several limitations should be acknowledged. First, while the GWAS identifies genomic regions associated with phenotypic traits, it does not establish causality; therefore, the candidate genes proposed here require experimental validation through expression analyses, functional genomic studies, and in vitro or in vivo assays. Second, the study population consisted exclusively of WB chickens at 43 weeks of age, and the genetic architecture of eggshell quality may differ across laying stages and breeds [8]. Third, the sample size was relatively small (n = 449), which may have limited the statistical power to detect loci with small effect sizes. Cross-population validation is required before the practical application of marker-assisted selection based on these findings. Future studies should include expression quantitative trait locus (eQTL) analyses and transcriptomic profiling of the shell gland to further elucidate the regulatory mechanisms by which the identified candidate genes influence eggshell quality traits.

## 5. Conclusions

In summary, a GWAS was used to reveal the genetic architecture of eggshell quality in Chinese Wenshang Barred chickens. We detected 87 SNPs and 14 candidate genes associated with eggshell quality. Ultimately, eleven genes, *MC2R*, *CDC73*, *CYP19A1*, *TNFAIP8L3*, *DMD*, *PHEX*, *ATM*, *SLIT3*, *RALY*, *SOCS5*, and *ATG4C*, were considered the most promising genes associated with eggshell quality, and they were implicated in estrogens, calcium transport, and phospholipid metabolism, while their function in laying hens requires further studies. This study provides a reference for further exploring the molecular mechanisms underlying egg quality.

## Figures and Tables

**Table 1:** Eggshell quality description at 43 weeks.

Trait	*n*	Mean	SD	CV (%)	Min	Max
ESW (g)	449	5.01	0.46	9.20	3.55	6.58
EST (mm)	449	0.41	0.05	11.44	0.24	0.52
ESS (N/cm^2^)	449	39.79	7.10	17.84	15.91	55.89

N: number; Mean: arithmetic mean; SD: standard deviation; CV: coefficient of variation; ESW: eggshell weight; EST: eggshell thickness; ESS: eggshell strength.

**Table 2:** Genome-wide significant SNPs associated with eggshell quality traits.

Trait	Chr	Base-Pair Region		nSNP	Related Genes
		Start	End		
ESW	2	96,507,469	-	1	*MC2R*
ESW	3	21,773,060	21,989,996	13	-
ESW	6	4,313,157	-	1	-
ESW	8	2,838,976	3,729,939	14	*CFH*/*CDC73*
ESW	10	9,530,698	9,902,841	26	*LYSMD2*/*TNFAIP8L3*/*CYP19A1*
ESW	23	4,710,746	4,718,567	2	-
EST	1	116,212,599	181,607,182	7	*DMD*/*PHEX*/*ATM*
EST	3	28,603,715	-	1	-
EST	4	52,072,388	52,696,520	2	-
EST	5	32,696,784	-	1	*STXBP6*
EST	6	4,566,470	4,721,806	2	-
EST	13	4,955,035	-	1	*SLIT3*
EST	20	1,789,304	1,981,793	4	*RALY*
ESS	3	26,912,693	26,961,126	10	*SOCS5*
ESS	8	27,290,593	279,647,37	2	*ATG4C*

Note: ESW: eggshell weight; EST: eggshell thickness; ESS: eggshell strength; Chr: chromosome; Base-pair region: physical position; nSNP: number of SNPs in base-pair region.

**Table 3:** GWAS and annotations of lead SNPs for eggshell quality traits.

Traits	Chr	Ps	SNP	Allele	Af	Beta ± Se	*p*	PVE
ESW	3	21,983,632	Chr3:21983632	G/C	0.21	−0.20 ± 0.04	1.04 × 10^−7^	6.12%
ESW	10	9,674,599	rs80641237	C/A	0.29	−0.17 ± 0.03	1.69 × 10^−7^	5.92%
ESS	3	26,940,075	rs731402681	G/A	0.44	2.50 ± 0.47	2.11 × 10^−7^	5.83%

Note: ESW: eggshell weight; ESS: eggshell strength.

## Data Availability

The genome sequence data reported in this article was uploaded to the genome sequence file of the BIG Data Center of the Beijing Institute of Genomics, Chinese Academy of Sciences, and is publicly available at http://bigd.big.ac.cn (CRA011183), accessed on 7 July 2026.

## Supplementary Materials

The following supporting information can be downloaded at https://www.mdpi.com/article/10.3390/ani16142157/s1, Table S1: The clean data and mapping details of resequencing samples; Table S2: GWAS results of suggestive SNPs for eggshell quality traits; Table S3: Annotations of suggestive SNPs for the eggshell quality traits.

## Abbreviations

The following abbreviations are used in this manuscript:

WB	Chinese Wenshang Barred chickens
ESW	Eggshell weight
EST	Eggshell thickness
ESS	Eggshell strength
PVE	Phenotypic variation explained
SNP	Single-nucleotide polymorphism
GGA	Chromosome
LD	Linkage disequilibrium
